# Melatonin disturbed rumen microflora structure and metabolic pathways *in vitro*

**DOI:** 10.1128/spectrum.00327-23

**Published:** 2023-11-06

**Authors:** Chun Xue, Yifan Wang, Zhaoyuan He, Zhiqi Lu, Feifan Wu, Yusu Wang, Yongkang Zhen, Jimeng Meng, Khuram Shahzad, Kailun Yang, Mengzhi Wang

**Affiliations:** 1 College of Animal Science and Technology, Yangzhou University, Yangzhou, China; 2 State Key Laboratory of Sheep Genetic Improvement and Healthy Production, Xinjiang Academy of Agricultural Reclamation Sciences, Shihezi, China; 3 College of Animal Science and Technology, Guangxi University, Nanning, China; 4 Ningxia Dairy Science and Innovation Center of Guangming Animal Husbandry Co., Ltd., Zhongwei, China; 5 Department of Biosciences, COMSATS University Islamabad, Islamabad, Pakistan; 6 College of Animal Science, Xinjiang Agricultural University, Urumqi, China; Jilin University, Changchun, China

**Keywords:** melatonin, rumen microflora, metabolomics, enzyme activity

## Abstract

Melatonin (MLT) can affect the microbial community structure and its metabolites by releasing cellular regulatory factors. Intestinal bacteria recognize and respond to MLT signals from the intestine through MLT binding sites and further activate the function of intestinal immune cells. However, the signal transduction pathway of MLT in the intestine has not been clearly elucidated yet. The effect of MLT on rumen bacterial metabolism was studied by simulating rumen fermentation *in vitro*. The test was divided into three groups: artificial saliva+standard MLT solution (CK0 group), artificial saliva+rumen juice (CK1 group), and artificial saliva+rumen juice+standard MLT solution (MLT group). The fermentation broth was collected after 24 h of simulated culture *in vitro*. The results showed that the addition of MLT significantly increased the contents of propionic acid, butyric acid, valeric acid, and total volatile fatty acids (*P <* 0.05). The analysis of the differences in flora found that at the genus level, *Alistipes*, *Veillonellaceae_UCG-001*, *Selenomonas*, *Lachnospiraceae_NK4A136_group,* and *Succinivibrio* were significantly increased, whereas *Acinetobacter* and *Prevotella* were significantly decreased (*P <* 0.05). The analysis of differential metabolites showed that compared with the CK1 group, the relative contents of glutathione, spermidine, indole, 3-methyl indole, phenylpyruvate, and other metabolites were increased significantly in the MLT group (*P <* 0.05). The results of enzyme activity on the enrichment pathway showed that MLT could significantly enhance the activities of genes such as GST, SPDS, DAHP, AS, TS, TPH, AANAT, ASMT, and IDO. Therefore, melatonin had certain regulatory effects on rumen microorganisms and their metabolic pathways.

**IMPORTANCE:**

In *in vitro* studies, it has been found that the effects of MLT on rumen microorganisms and metabolites can change the rumen flora structure, significantly inhibit the relative abundance of harmful *Acinetobacter,* and improve the relative abundance of beneficial bacteria. MLT may regulate the “arginine-glutathione” pathway, “phenylalanine, tyrosine and tryptophan biosynthesis-tryptophan generation” branch, “tryptophan-kynurenine” metabolism, and “tryptophan-tryptamine-serotonin” pathway through microorganisms.

## INTRODUCTION

Melatonin (MLT), an amine hormone, can be synthesized and secreted not only in the pineal gland of the hypothalamic suprachiasmatic nucleus (1) but also in cells (2) and the gastrointestinal tract (3). Enterochromaffin cells in the gastrointestinal tract can secrete melatonin. Among these, MLT from the gastrointestinal tract can regulate gastrointestinal movement (4), improve antioxidant capacity (5), and increase immune regulation in the body (6). The metabolism of MLT in the liver is the main metabolic pathway out of the three main MLT metabolic pathways. MLT can be converted into 6-hydroxymelatonin under the action of the cytochrome P540 family, e.g., *CYP1A1*, *CYP1A2*, and *CYP1B1*. 6-Hydroxymelatonin can be metabolized to glucuronide and sulfate metabolites by hydroxylation. It can also be demethylated to generate N-acetylserotonin (NAS), NAS glucoside, and NAS sulfate (7). The production of 5-methylindoleacetic acid or 5-methyltryptophan from MLT is a secondary pathway of MLT metabolism. The last pathway of MLT metabolism is the kynuric acid pathway, in which N1-acetyl-5-methoxykynurenine is the final product. MLT can be used as a signal molecule to participate in the communication between intestinal microorganisms and to affect the microbial community structure and its metabolites by releasing cellular regulatory factors (7). Intestinal bacteria recognize and respond to MLT signals through intestinal MLT binding sites and further activate intestinal immune cells (8, 9). Previous studies (10) have found that in male mice that ingested MLT by gavage, the ratio of *Firmicutes* and *Bacteroidetes* in the gut was significantly reduced, and the relative abundance of *Akkermansia* was increased. The reduction in gut bacterial quantity and diversity suggested that MLT may suppress obesity in mice by altering the structure of the gastrointestinal microbiota and had a positive effect on regulating insulin resistance and low-grade inflammation.

Tryptophan (Trp) plays an important role in maintaining intestinal immune tolerance and the balance of gut microbiota. A previous study has found that along with MLT, Trp and its related metabolites, such as tryptamine, indole acid, and indole, have a huge impact on microbial metabolism (11). A study about the effects of MLT on the gut microbiota showed that gut microbes can determine the effects of Trp on the body through pronounced changes in amino acid metabolites, such as bacteria that produce indole sulfate and indole-3-propionic acid (IPA) (12). The formation of IPA is entirely dependent on gut microbes and can be enhanced by colonization with *Clostridium sporogenes*. MLT affects the intestinal microbial composition by regulating intestinal microorganisms and their metabolism through the NF-κB channel (13). However, the MLT signal transduction pathways in the rumen are unknown. The microorganisms that mediate changes in these metabolic pathways are also not known. Therefore, we proposed a hypothesis that MLT had a direct or indirect impact on changes in rumen microbiota and metabolites.

In our study, mixed rumen microorganisms were cultured by *in vitro* culture technology along with MLT to explore the effects on rumen microbial flora and metabolic reactions.

## MATERIALS AND METHODS

### Collection of rumen fluid

Three 1.5-year-old Hu sheep with permanent rumen fistula were randomly selected from the experimental pasture of Yangzhou University. The animals were fed total mixed ration (TMR) feed twice a day. Fifty milliliters of rumen fluid was collected from each Hu sheep before feeding at 8 o’clock in the morning and filtered with four layers of gauze. Subsequently, it was placed in a thermos (the thermos was filled with CO_2_ in advance and kept warm with 39°C warm water) immediately and then brought back to the laboratory to keep the rumen fluid in the 39°C water baths, with a continuous supply of CO_2_ until artificial rumen fluid was prepared.

### *In vitro* fermentation device

The *in vitro* fermentation device was composed of a SHA-A constant temperature shaking water bath and 250-mL fermentation flasks. The rumen fluid was incubated at 39°C for 24 h and continuously fed CO_2_. The fluid was collected after 24 h.

### Preparation of artificial saliva and standard MLT solution

To prepare artificial saliva, the reagents were added, as shown in Table 1. The saliva was fully mixed, CO_2_ was continuously injected for about 15 min, and it was placed in a water bath at 39°C. Artificial saliva was prepared by mixing the reagents shown in Table 1, CO_2_ was injected into the solution for 15 min, and it was finally placed in a water bath at 39°C. The stock solution of MLT was prepared by dissolving 0.9 mg of MLT in a trace amount of ethanol, and ultrapure water was used to make a final volume of 100 mL. One milliliter of MLT stock solution was taken and diluted 1,000 times to prepare a 9 ng/mL standard MLT solution. The final concentration of MLT was prepared to 50 µg/mL. The *in vitro* fermentation batch is shown in Table 2.

**TABLE 1:** Artificial saliva allocation (1,000 mL)

Chemical reagent	Requirement
NaHCO_3_	8.75 g
NH_4_HCO_3_	1.00 g
Na_2_HPO_4_	1.43 g
MgSO_4_·7H_2_O	0.1581 g
Na_2_S	0.52 g
MnCl_2_·4H O	0.015 g
CoCl_2_·6H_2_O	0.002 g
FeCl_3_·6H_2_O	0.012 g
KH_2_PO_4_	1.55 g
CaCl_2_·2H_2_O	0.017 g
Resazurin	1.25 mg

**TABLE 2:** *In vitro* fermentation batch culture test groups^*a*^

Item	Groups		
	CK0	CK1	MLT
Artificial saliva	119 mL	119 mL	119 mL
Rumen fluid	/^*b*^	60 mL	60 mL
MLT standard solution	1 mL	/	1 mL
Ultrapure water	60 mL	1 mL	/

^*a*^ CK0, artificial saliva+MLT standard solution; CK1, artificial saliva+rumen fluid; MLT, artificial saliva+rumen fluid+MLT standard solution.

^*b*^ /, without adding.

### Determination of MLT concentration

To understand the consumption of melatonin, the concentration of MLT in all samples was measured (North Institute of Biotechnology in Beijing, China) by radioimmunoassay (RIA) for detection.

### Determination of fermentation parameters of rumen fluid

A Shanghai Leici pHS-3C precision pH meter was used to measure the pH of fermented rumen fluid. Before measurement, acid and neutral correction solutions were used to correct the pH meter. The concentration of NH_3_-N was measured by a 756 visible ultraviolet spectrophotometer. The content of mycoprotein (MCP) was determined by the purine base method. The content of volatile fatty acid (VFA) was analyzed by a Shimadzu GC-14B gas chromatograph in Japan.

### DNA extraction, 16S rRNA gene amplification, sequencing, and analysis

According to the manufacturer’s 16S rRNA sequencing instructions, total microbial DNA from samples from the CK1 and MLT groups was extracted using the FastPure Bacteria DNA Isolation Mini Kit (DC103, Vazyme Biotechnology Co., Ltd., Nanjing, China). The concentration and purity of DNA were detected by an ultramicro spectrophotometer (NanoDrop-1000, SEM Fisher, USA), and the quality was checked by agarose gel electrophoresis. The DNA was stored in a −20°C refrigerator until high-throughput sequencing experiments. Next-generation sequencing library preparations and Illumina MiSeq sequencing were conducted at Novogene Inc. (Nanjing, China). The sequencing system used was a HiSeq PE 250 sequencing platform. The bacterial 16S rRNA gene was amplified by universal primers (319F: 50-ACTCCTACGGGAGGCAGCAG-30; 806R: 50-GGACTACHVGGGTWTCTAAT-30) in the V3-V4 region. All PCRs were performed with 15 µL of Phusion High-Fidelity PCR Master Mix (New England Biolabs). Two-micromolar forward and reverse primers were used, along with approximately 10 ng of template DNA. Thermal cycling consisted of 30 cycles of initial denaturation at 98°C for 1 min, followed by denaturation at 98°C for 10 s, annealing at 50°C for 30 s, and extension at 72°C for 30 s, finally, 72°C for 5 min. Sequencing libraries were generated using a TruSeq DNA PCR-Free Sample Preparation Kit (Illumina, USA) by following the manufacturer’s recommendations. Index codes were added. The library quality was assessed on a Qubit@ 2.0 Fluorometer (Thermo Scientific) and Agilent Bioanalyzer 2100 system. Finally, the library was sequenced on an Illumina NovaSeq platform, and 250-bp paired-end reads were generated (14). The raw labels were then quality filtered under specific filtering conditions according to the quality control process of QIIME (V1.9.1) to obtain high-quality clean labels (15). Sequence analysis was performed using UPARSE software (UPARSE V7.0.1001). Sequences with ≥97% similarity were assigned to the same operational taxonomic units (OTUs). A representative sequence for each OTU was screened for further annotation (16). For each representative sequence, the SILVA database was used based on the Mothur algorithm to annotate taxonomic information (17). Six indices were analyzed for alpha diversity, including observed species, Chao1, Shannon, Simpson, ACE, and good coverage, and were calculated with QIIME (V1.9.1) and displayed using R software (version 2.15.3). The beta diversity using weighted and unweighted UniFrac was calculated using QIIME software (version 1.9.1).

### Metabolomics processing and analysis

Every rumen fluid sample (100 µL) in the CK1 and MLT groups and pre-cooled methanol (400 µL) were mixed by thorough vortexing (18). Samples were placed in an ice bath for 5 min and then centrifuged at 15,000 rpm for 10 min at 4°C. A portion of the supernatant was diluted with high performance liquid chromatography (HPLC) grade water to a final concentration containing 53% methanol. The sample was then transferred to a clean Eppendorf tube and centrifuged at 15,000 rpm for 10 min at 4°C. Finally, the supernatant was injected into the ultra-high performance liquid chromatography-mass spectrometry (UHPLC-MS/MS) system for analysis (19). UHPLC-MS/MS analyses were performed using a Vanquish UHPLC system (Thermo Fisher, Germany) coupled with an Orbitrap Q Exactive HF mass spectrometer (Thermo Fisher, Germany) at Novogene Co., Ltd. (Beijing, China). The eluents in the positive polarity mode contained eluent A (0.1% FA in water) and eluent B (methanol). The eluents for the negative polarity mode included eluent A (5 mM ammonium acetate, pH 9.0) and eluent B (methanol). The solvent gradient was set as follows: 2% B, 1.5 min; 2%–100% B, 12.0 min; 100% B, 14.0 min; 100%–2% B, 14.1 min; and 2% B, 17 min. The Q Exactive HF mass spectrometer was operated in positive/negative polar mode with a spray voltage of 3.2 kV, capillary temperature of 320 ℃, intrathecal gas flow rate of 40 arbitrary units (arb), and an auxiliary gas flow rate of 10 arb.

Raw data files generated by ultra-performance liquid chromatography-MS/MS were processed with Compound Discoverer 3.1 (CD3.1, Thermo Fisher) for peak alignment, peak picking, and quantitative analysis of each metabolite. The main parameters were set as follows: retention time tolerance, 0.2 min; actual mass tolerance, 5 ppm; signal intensity tolerance, 30%; signal/noise ratio, 3; and minimum intensity, 100,000. The intensity of the peaks was normalized to the total spectral intensity. The normalized data were used to predict molecular formulae based on addition ions, molecular ion peaks, and fragment ions. These peaks were later matched against the mzCloud (https://www.mzcloud.org/), mzVault, and MassList databases for accurate qualitative and relative quantitative results. Statistical analyses were performed using the statistical software R (R version R-3.4.3), Python (Python 2.7.6 version), and CentOS (CentOS release 6.6). The data were processed using the normalization method. Principal component analysis (PCA) was performed using metaX (a flexible and comprehensive software for processing metabolomics data). We applied univariate analysis of variance (*t*-test) to calculate statistical significance (*P*-value). The metabolites with variable importance in the projection (VIP) > 1 and *P*-value < 0.05 and fold change (FC) ≥ 2 or FC ≤ 0.5 were considered differential metabolites. Volcano plots were used to screen metabolites of interest based on log2 (fold change) and -log10 m (*P*-value).

### Determination of enzyme activity

Indoleamine 2,3-dioxygenase (IDO), acetylserotonin-o-methyltransferase (ASMT), spermidine synthase (SPDS), Try hydroxylase (TPH), Try synthase (TS), o-aminobenzoic acid synthase (AS), glutathione-S-transferase (GST), aromatic amine-n-acetyltransferase (AANAT), and 3-deoxy-d-arabinose-heptanoic acid-7-phosphate synthase (DAHP) were analyzed using ELISA kits, which were purchased from Shanghai Enzyme-linked Biotechnology Co., Ltd. Two milliliters of rumen fluid was homogenized and centrifuged at 4°C at 5,000 rpm for 8 min, and the supernatant was taken for enzyme activity determination by following the specific manual’s instructions for each enzyme.

### Data analysis

The data for MLT concentration in the culture medium and the results of fermentation parameters were sorted before applying statistical analysis using SPSS 25.0 software. Two-way ANOVA was used for MLT concentration, and a *t*-test was used for rumen fermentation parameters. *P <* 0.05 was considered a significant difference. Similarly, the data of metabolomics experiments were preliminarily sorted before applying the statistical analysis. The GraphPad statistics tool was used for data visualization. Multivariate statistical analysis-based PCA was used, and then orthogonal projections to latent structures discriminant analysis (OPLS-DA) was used to filter out orthogonal variables that were not related to categorical variables in metabolites. The orthogonal and non-orthogonal variables were analyzed separately. Finally, combined with the *t*-test and OPLS-DA, the differential metabolites were screened out and analyzed.

## RESULTS

### The results of MLT concentration

As shown in Table 3, the treatment method significantly affected the MLT concentration. After 24 h, the concentration of MLT in the CK0 group was significantly higher than the CK1 group and significantly lower than the MLT group. The degradation rate of MLT in the CK0 group was significantly lower than that in the CK1 and MLT groups (*P* < 0.05).

**TABLE 3:** Changes in MLT concentration

Time	Treatment			SEM	STD
	CK0	CK1	MLT		
0 h	48.17^*b*^	50.39^*b*^	99.83^*a*^	1.39	2.41
24 h	28.08^*a*^ ^,*b*^	15.79^*b*^	57.82^*a*^		
Degradation rate (pg·mL^−1^·h^−1^)	0.84^*b*^	1.44^*a*^	1.75^*a*^		

^*a*^ Means within a row with different letters differ significantly (*P* < 0.05).

^*b*^ Means within a row with different letters differ significantly (*P* < 0.05).

### Rumen fermentation parameters

The pH, NH_3_-N, and MCP had no significant changes after rumen fermentation *in vitro* for 24 h, as shown in Table 4. The concentrations of propionic acid, isobutyric acid, butyric acid, valeric acid, and total volatile fatty acid (TVFA) were increased significantly after 24 h of fermentation with MLT in rumen fluid (*P* < 0.05), as shown in Table 4. The ratio of ethyl to propylene decreased significantly (*P* < 0.05). The acetic acid to propionic acid ratio was <2.5, indicating that MLT changed the rumen fermentation type, made the rumen fermentation develop to propionic acid type, and provided more energy for the body.

**TABLE 4:** Changes in rumen levels of NH3-N, MCP, pH, and VFA

Item		Groups		*P*-value
		CK1	MLT	
NH_3_-N	24 h	13.76 ± 0.78	13.38 ± 0.45	0.698
MCP	24 h	0.12 ± 0.02	0.13 ± 0.35	0.506
pH	24 h	6.93 ± 0.03	6.90 ± 0.02	0.508
Acetic acid	24 h	15.07 ± 0.53	14.69 ± 0.45	0.392
Propionic acid	24 h	13.83 ± 0.28	14.99 ± 0.19	0.004
Isobutyric acid	24 h	7.23 ± 0.12	7.71 ± 0.19	0.023
Butyric acid	24 h	8.35 ± 0.07	9.40 ± 0.24	0.002
Isovaleric acid	24 h	2.13 ± 0.14	2.41 ± 0.11	0.05
Pentanoic acid	24 h	1.05 ± 0.04	1.20 ± 0.07	0.033
TVFA	24 h	47.66 ± 0.75	50.40 ± 1.11	0.024
Acetic acid/pentanoic acid	24 h	1.09 ± 0.04	0.98 ± 0.02	0.01

### Rumen microflora profile

#### Statistics of the sequence number and OTU number

**Fig 1:**
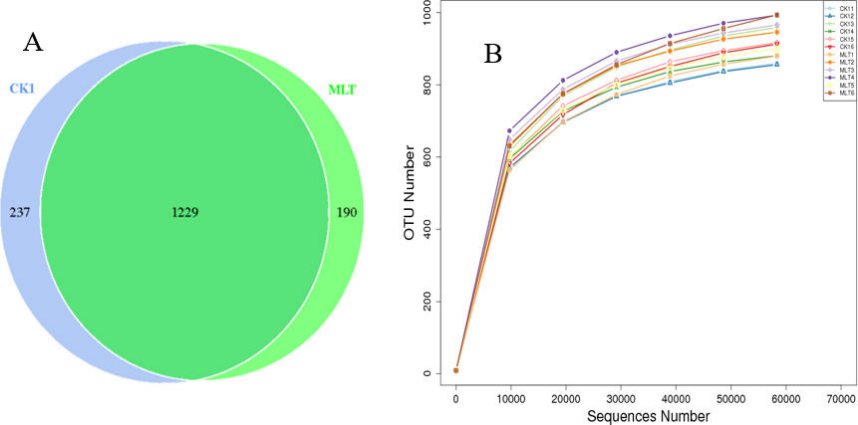
Ruminal microbial operational taxonomic units (OTUs) with different treatments of adding MLT. CK1, artificial saliva+rumen fluid; MLT, artificial saliva+rumen fluid+standard MLT solution. (**A**) Venn diagram of ruminal bacterial OTUs. (**B**) Bacterial rarefaction curves based on OTUs were used to assess the depth of coverage for each sample.

OTUs with 97% identity were clustered by valid sequences from all samples, and the sequences of OTUs were then species annotated. Then, we extracted a certain amount of sequencing data from the samples, counted the number of OTUs, and constructed a dilution curve according to the amount of extracted sequencing data and the corresponding number of species. The results are shown in Fig. 1A and B. These results indicated that a total of 1,656 OTUs were detected in the samples. Among them, there were 1,229 repeated OTUs. The CK1 group had 237 unique OTUs, and the MLT group had 190 unique OTUs. The results showed that with the increased number of OTUs, the curves of all samples tended to be flat, indicating that the increase in sequencing depth no longer affected the identification of bacterial species, and the amount of sequencing was reasonable.

#### Alpha diversity analysis

Alpha diversity was used to analyze the diversity of microbial communities in the sample. Alpha diversity could reflect the richness and diversity of microbial communities. It can be seen from Table 5 that the Shannon index and Simpson index of the MLT group were significantly higher than those of the CK1 group. This indicated that the diversity and uniformity of species distribution in the MLT group were relatively high.

**TABLE 5:** Analysis of the alpha diversity index

Item	Groups		*P*-value
	CK1	MLT	
Shannon	6.25 ± 0.29	6.91 ± 0.34	0.004
Simpson	0.94 ± 0.03	0.97 ± 0.10	0.020
Chao1	951.56 ± 42.85	1,074.60 ± 193.04	0.158
ACE	956.60 ± 46.25	1,024.48 ± 76.62	0.093
Coverage	0.998 ± 0.00	0.998 ± 0.00	0.363

#### Beta diversity analysis

**Fig 2:**
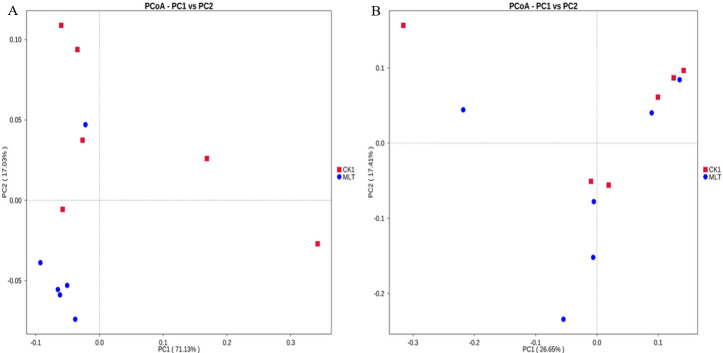
PCoA based on the weighted UniFrac distance (**A**) and unweighted UniFrac distance (**B**).

As shown in Fig. 2, principal coordinates analysis (PCoA) was performed based on the weighted UniFrac distance and the unweighted UniFrac distance. The difference contribution values of PC1 to the weighted UniFrac distance and the unweighted UniFrac distance of the samples are 71.13% and 17.03%. The difference contribution values of PC2 to the weighted UniFrac distance and the unweighted UniFrac distance of the samples are 26.65% and 17.41%. In the weighted UniFrac, the two groups of samples were not clearly separated, and there were overlapping parts, indicating that the bacterial composition of the two groups had similar and different parts. Unweighted UniFrac mainly analyzed the existence and evolution of species. The results showed that the species in the experimental group were more abundant than those in the CK1 group.

#### Microflora structure at the phylum level

The bacteria in the samples from the CK1 and MLT groups were mainly classified into three categories: *Bacteroidota*, *Firmicutes,* and *Proteobacteria,* as shown in Table 6. The results showed that *unidentified_Bacteria* and *Verrucomicrobiota* were significantly higher and *Proteobacteria* and *Euryarchaeota* were significantly lower in the MLT group as compared with the CK1 group (*P* < 0.05).

**TABLE 6:** Distribution of the top 10 microflora at the phylum level

Item	Groups		*P*-value
	CK1	MLT	
Bacteroidota	0.394 ± 0.115	0.456 ± 0.018	0.218
Firmicutes	0.281 ± 0.069	0.303 ± 0.026	0.490
Proteobacteria	0.265 ± 0.071	0.176 ± 0.027	0.016
Unidentified_Bacteria	0.029 ± 0.005	0.039 ± 0.004	0.003
Spirochaetota	0.010 ± 0.012	0.005 ± 0.001	0.361
Verrucomicrobiota	0.003 ± 0.001	0.008 ± 0.003	0.004
Euryarchaeota	0.005 ± 0.003	0.001 ± 0.001	0.026
Actinobacteriota	0.003 ± 0.001	0.003 ± 0.001	0.802
Desulfobacterota	0.002 ± 0.001	0.001 ± 0.000	0.820
Deferribacteres	0.000 ± 0.000	0.001 ± 0.000	0.120

#### Microflora structure at the genus level

The top 30 bacterial genera at the genus level are shown in Table 7, among which the dominant bacterial genera of the MLT group and the CK1 group were *Rikenellaceae_RC9_gut_group* and *Acinetobacter,* respectively. The relative abundances of *Acinetobacter*, *Prevotellaceae_UCG-004*, and *Methanobacter* in the MLT group were significantly lower than those in the CK1 group (*P <* 0.05). The relative contents of *Lacetospiraceae_NK4A136_group*, *Alistipes*, *Veillonellaceae_UCG-001*, *Semenomonas*, and *Succinivibrio* were significantly higher than those of the CK1 group (*P* < 0.05). In addition, the results showed that the experimental fistula Hu sheep may be infected with *Acinetobacter* during the feeding process. However, the relative abundance of *Acinetobacter* was significantly reduced after adding MLT to the fermentation broth (*P <* 0.05). The increase in the relative content of some bacteria, such as *Laurospirillaceae_NK4A136_group*, *Rumenobacter*, and *Akkermansia,* may also lead to a decrease in the relative content of *Acinetobacter*.

**TABLE 7:** Distribution of the top 30 microflora at the genus level

Item	Groups		*P*-value
	CK1	MLT	
Acinetobacter	0.256 ± 0.073	0.163 ± 0.030	0.016
Rikenellaceae_RC9_gut_group	0.200 ± 0.078	0.206 ± 0.019	0.860
Prevotella	0.042 ± 0.017	0.041 ± 0.017	0.897
Succiniclasticum	0.017 ± 0.008	0.017 ± 0.004	0.909
Treponema	0.007 ± 0.012	0.003 ± 0.000	0.311
Pseudobutyrivibrio	0.017 ± 0.009	0.011 ± 0.002	0.129
FD2005	0.012 ± 0.009	0.017 ± 0.003	0.310
Christensenellaceae_R-7_group	0.021 ± 0.003	0.020 ± 0.002	0.585
NK4A214_group	0.013 ± 0.005	0.013 ± 0.002	0.868
Lactobacillus	0.003 ± 0.001	0.008 ± 0.006	0.105
Lachnospiraceae_NK4A136_group	0.005 ± 0.002	0.004 ± 0.003	0.002
Alistipes	0.003 ± 0.001	0.009 ± 0.033	0.002
Prevotellaceae_UCG-004	0.005 ± 0.002	0.005 ± 0.001	0.000
Ruminococcus	0.007 ± 0.002	0.006 ± 0.002	0.573
Veillonellaceae_UCG-001	0.002 ± 0.001	0.008 ± 0.003	0.002
Prevotellaceae_UCG-001	0.006 ± 0.001	0.007 ± 0.002	0.132
Methanobrevibacter	0.005 ± 0.003	0.001 ± 0.001	0.026
Selenomonas	0.003 ± 0.001	0.007 ± 0.002	0.002
Anaerovorax	0.007 ± 0.002	0.006 ± 0.000	0.322
UCG-002	0.003 ± 0.001	0.007 ± 0.002	0.000
Succinivibrio	0.004 ± 0.002	0.007 ± 0.001	0.005
Akkermansia	0.002 ± 0.001	0.005 ± 0.003	0.017
Ruminobacter	0.001 ± 0.001	0.005 ± 0.002	0.002
Candidatus_Saccharimonas	0.004 ± 0.002	0.003 ± 0.001	0.515
Saccharofermentans	0.003 ± 0.001	0.002 ± 0.001	0.119
Lachnospiraceae_NK3A20_group	0.004 ± 0.001	0.004 ± 0.003	0.706
Acetitomaculum	0.003 ± 0.001	0.003 ± 0.001	0.596
Sphaerochaeta	0.003 ± 0.002	0.004 ± 0.001	0.109
Kerstersia	0.001 ± 0.002	0.000	0.363
Anaerovibrio	0.002 ± 0.001	0.002 ± 0.001	0.550

### Rumen metabolomics profiling

#### Multivariate statistical analysis

**Fig 3:**
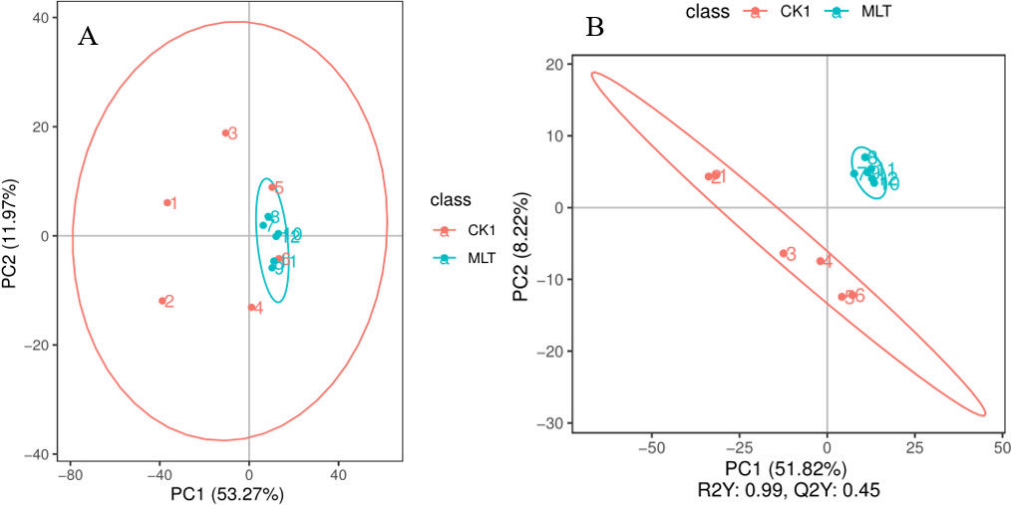
PCA score diagram. The abscissa PC1 and ordinate PC2 represent the principal component scores of the first ranking and the second ranking, respectively, and the ellipse is the 95% confidence interval (A). PLS-DA score chart. The abscissa is the score of the sample on the first principal component (B). The ordinate is the score of the sample on the second principal component. R2Y indicates the interpretation rate of the model, Q2Y is used to evaluate the prediction ability of the PLS-DA model, Q2Y is used to evaluate the prediction ability of the PLS-DA model, and R2Y greater than Q2Y indicates that the model is well established.

First, PCA was performed in the CK1 group and MLT group to observe the change trend of the two groups. As shown in Fig. 3A, the samples in the MLT group are clustered, and the CK1 samples were scattered. The intragroup difference between the MLT group and CK1 group was small, and all samples were within the 95% confidence interval. Partial least squares discriminant analysis (PLS-DA) was performed for the two groups. The results are shown in Fig. 3B. The samples of the CK1 group and MLT group were significantly distinguished, and the samples of the MLT group were more clustered. According to the results of PLS-DA, two principal components, such as R2Y with 0.99 and Q2Y with 0.45, were obtained from the sample. The results indicated that the fitting degree and prediction ability of the model are ideal, and the model is stable, reliable, and of good quality.

#### Screening of differential metabolites

**Fig 4:**
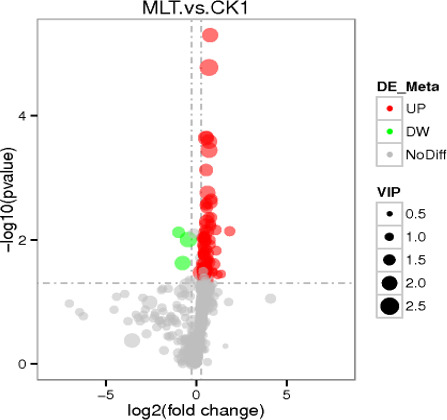
Differential metabolite volcano map. Each point in the volcanic map represents a metabolite. The significantly upregulated metabolites are represented by red points, and the significantly downregulated metabolites are represented by green points.

The screening of differential metabolites mainly referred to the three parameters of VIP, FC, and *P*-value, where VIP > 1 and *P* < 0.05, and the screening results are shown in Fig. 4. Through qualitative and relative quantitative analyses of the screened differential metabolites, a total of 107 significantly different metabolites were identified. Among them, 104 differential metabolites were significantly upregulated, and three differential metabolites were significantly downregulated.

The *P*-value and VIP value of 107 significantly different metabolites were further analyzed (*P* < 0.05, VIP > 1). The smaller the *P*-value of different metabolites and the larger the VIP, the more significant metabolites were observed. We speculated that these differential metabolites were significantly related to the metabolism of MLT. The top 19 significantly different metabolites are shown in Table 8.

**TABLE 8:** Significant differences in metabolites^*a*^

Differential metabolites	FC	*P*-value	VIP	Trend
Spermidine	1.436	0.003	1.365	Up
Acetophenone	1.494	0.003	1.437	Up
Skatole	1.619	0.004	1.338	Up
4-Hydroxybenzoic acid	0.510	0.008	1.501	Down
D-Ala-D-Ala	0.738	0.010	2.184	Down
Estriol	1.269	0.013	1.135	Up
Glutathione	1.459	0.014	1.156	Up
TPH	1.271	0.017	1.072	Up
Nicotinuric acid	1.289	0.017	1.046	Up
Shikonin	1.484	0.022	1.027	Up
Biotin	1.516	0.022	1.095	Up
Arachidonic acid	1.462	0.023	1.036	Up
2,3-Dinor-8-epi-prostaglandin	1.269	0.023	1.056	Up
16(R)-HETE	1.258	0.024	1.481	Up
Xanthurenic acid	1.524	0.031	1.003	Up
Indole	1.893	0.033	1.035	Up
Phenylpyruvic acid	1.301	0.033	1.004	Up
Thymidine	1.556	0.033	1.002	Up
AMP	2.274	0.037	1.014	Up

^*a*^ VIP, variable importance in the projection; FC, fold change.

#### Metabolic pathway analysis of differential metabolites

**Fig 5:**
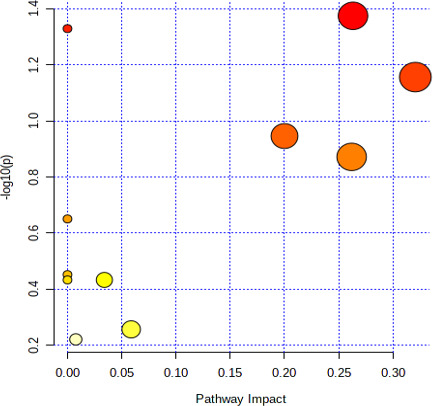
Pathway analysis results of differential metabolites.

The pathway analysis results in Fig. 5 and Table 9 are shown. These signaling pathways are mainly involved in (i) glutathione metabolism; (ii) phenylalanine, tyrosine, and Try biosynthesis; (iii) arachidonic acid metabolism; (iv) biotin metabolism; and (v) phenylalanine metabolism. The metabolic pathways contain different metabolites, as shown in Table 9. Glutathione and spermidine belonged to the glutathione metabolic pathway. Indole, TPH, and 4-hydroxybenzoic acid were the products of phenylalanine, tyrosine, and Try biosynthesis metabolism. TPH, xanthurenic acid, skatole, and indole were the products of the Try metabolic pathway.

**TABLE 9:** Analysis and classification of differential metabolite metabolic pathways

No.	Differential metabolites	Pathway	*P*-value
1	Glutathione and spermidine	Glutathione metabolism	0.042
2	Indole and TPH	Phenylalanine, tyrosine, and tryptophan biosynthesis	0.047
3	16(R)-HETE and arachidonic acid	Arachidonic acid metabolism	0.069
4	–^*a*^	Biotin metabolism	0.113
5	5-Hydroxybenzoic acid and phenylpyruvic acid	Phenylalanine metabolism	0.134

^*a*^ -, unavailable.

#### Correlation analysis of differential microorganisms and differential metabolites

**Fig 6:**
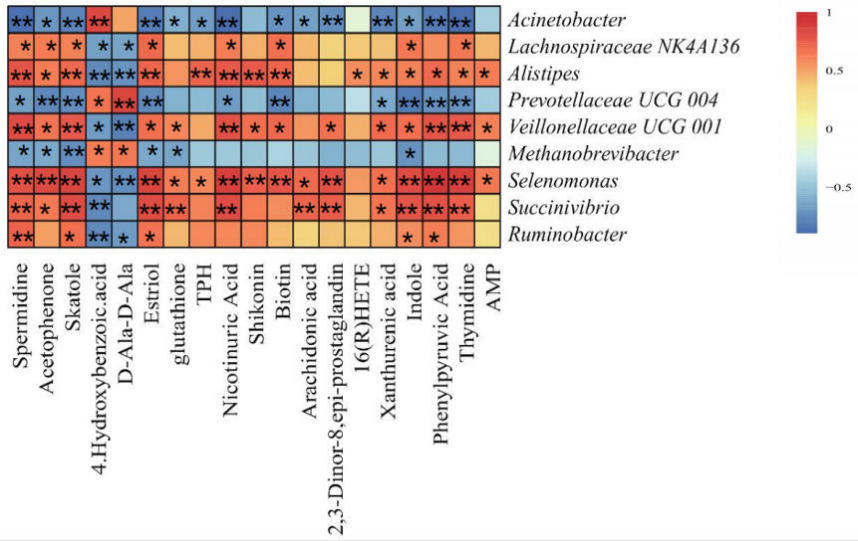
Correlation analysis between differential flora and differential metabolites. * means significant correlation, and ** means extremely significant correlation.

To further explore the relationship between rumen microbes and metabolites, Spearman’s analysis method was used to analyze the correlation between the differential microorganisms and the differential metabolites. The results are shown in Fig. 6. *Acinetobacter* showed a significant negative correlation with 14 metabolites and a significant positive correlation with 4-hydroxybenzoic acid (*P* < 0.05). There was a significant negative correlation between *Lachnospiraceae_NK4A136_Group* and two metabolites and a significant positive correlation with eight metabolites (*P* < 0.05). *Alistipes* had a significant negative correlation with two metabolites and a positive correlation with 14 metabolites (*P* < 0.05). *Prevotellaceae_UCG-00*4 was negatively correlated with 10 metabolites and positively correlated with two metabolites (*P* < 0.05). *Veillonellaceae_UCG-001* had a negative correlation with two metabolites and a significant positive correlation with 14 metabolites (*P* < 0.05). *Methanobrevibacter* had a significant negative correlation with six metabolites and a significant positive correlation with two metabolites (*P* < 0.05). *Selenomonas* had a significant negative correlation with two metabolites and a significant positive correlation with 16 metabolites (*P* < 0.05). *Succinivibrio* had a significant negative correlation with 4-hydroxybenzoic acid and a significant positive correlation with 12 metabolites (*P* < 0.05). *Ruminobacter* had a significant negative correlation with two metabolites and a significant positive correlation with five metabolites (*P* < 0.05).

**Fig 7:**
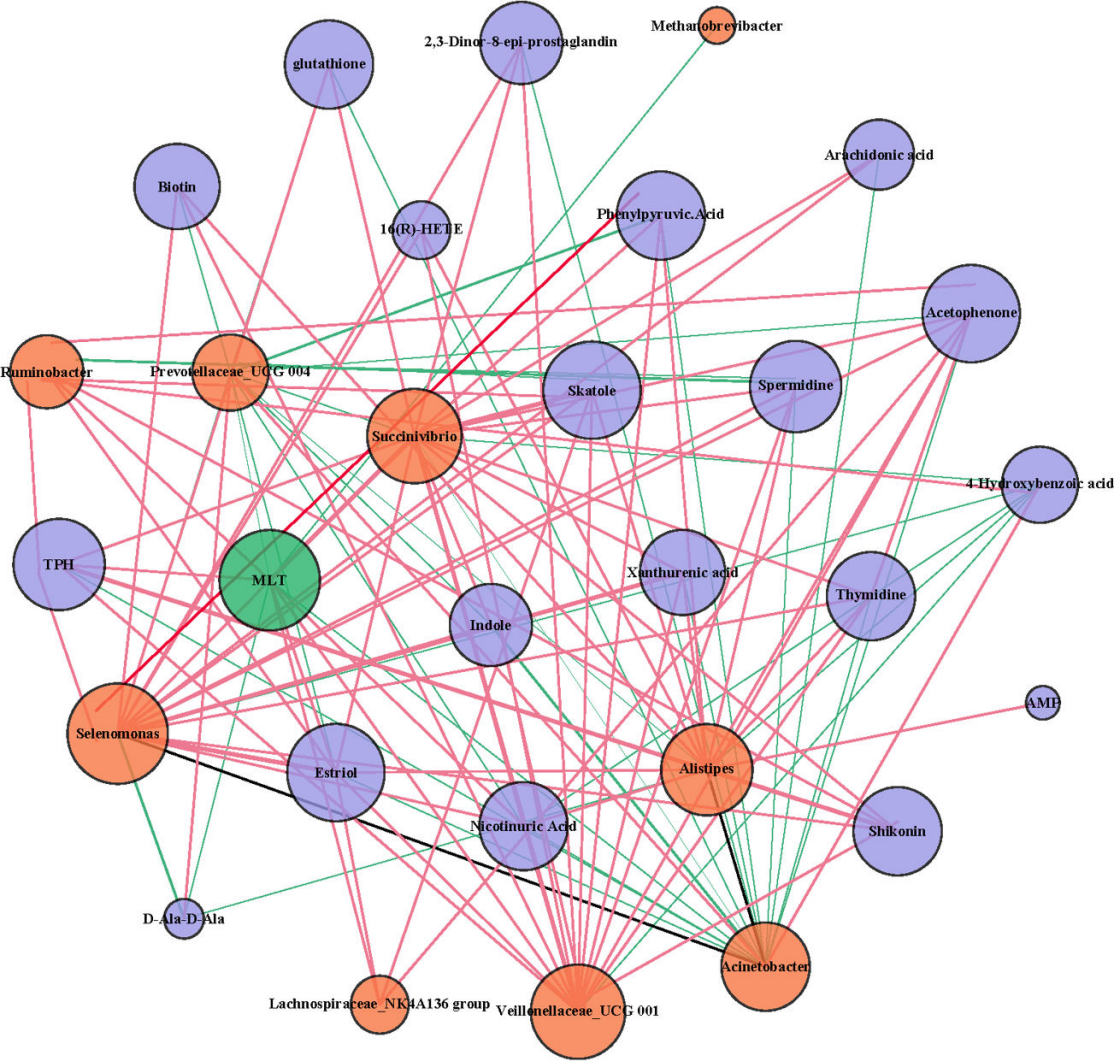
Network analysis between differential flora and differential metabolites. The red line represents a positive correlation, and the green line represents a negative correlation.

The results of network analysis (Fig. 7) showed that after the addition of MLT, there were three clusters of bacteria according to pathways: *Selenomonas*, *Succinivibrio*, *Veillonellaceae_UCG-001,* and *Alistipes,* which were related to the phenylalanine, tyrosine, and Try biosynthesis pathway. *Selenomonas*, *Succinivibrio*, *Veillonellaceae_UCG-001*, *Lachnospiraceae_NK4A136_group*, *Alistipes,* and *Ruminobacter* were related to the Try metabolic pathway. *Selenomonas*, *Succinivibrio*, *Veillonellaceae_UCG-001,* and *Alistipes* were related to the glutathione metabolic pathway.

### Determination of key enzyme activities and metabolites in the enrichment pathway

#### Effect of MLT on the activities of key enzymes in the enrichment pathway

**Fig 8:**
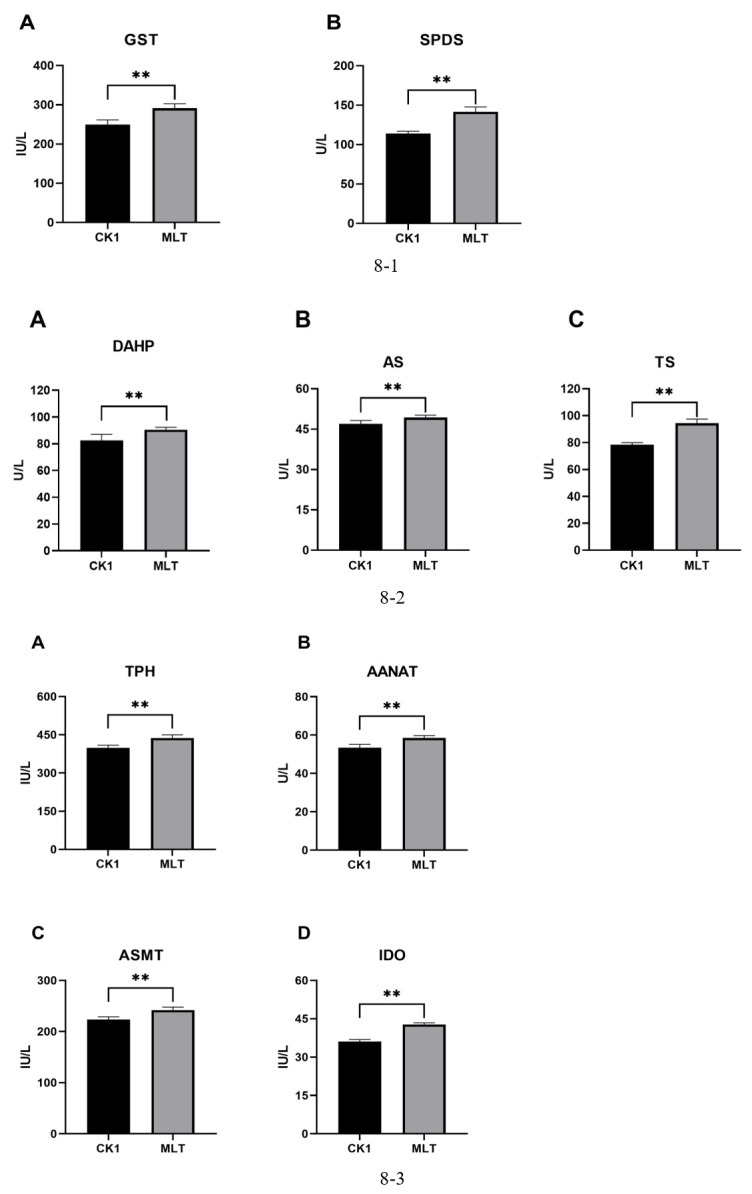
(1) Key enzymes in the glutathione pathway. * means significant correlation. (2) Key enzymes in the phenylalanine, tyrosine, and tryptophan biosynthesis pathway. * means significant correlation. (3) Key enzymes in the tryptophan metabolic pathway. * means significant correlation.

The enzyme activities of GST and SPDS (Fig. 8-1) in the glutathione metabolic pathway in the MLT group were significantly higher than those in the CK1 group (*P* < 0.05). In the phenylalanine, tyrosine, and Try biosynthesis pathway, the enzyme activities of DAHP, AS, and TS (Fig. 8-2) in the MLT group were significantly higher than those in the CK1 group (*P* < 0.05). In the Try metabolic pathway, the enzyme activities of TPH, AANAT, ASMT, and IDO (Fig. 8-3) in the MLT group were significantly higher than those in the CK1 group (*P* < 0.05).

#### Effect of MLT on metabolites in the enrichment pathway

**Fig 9:**
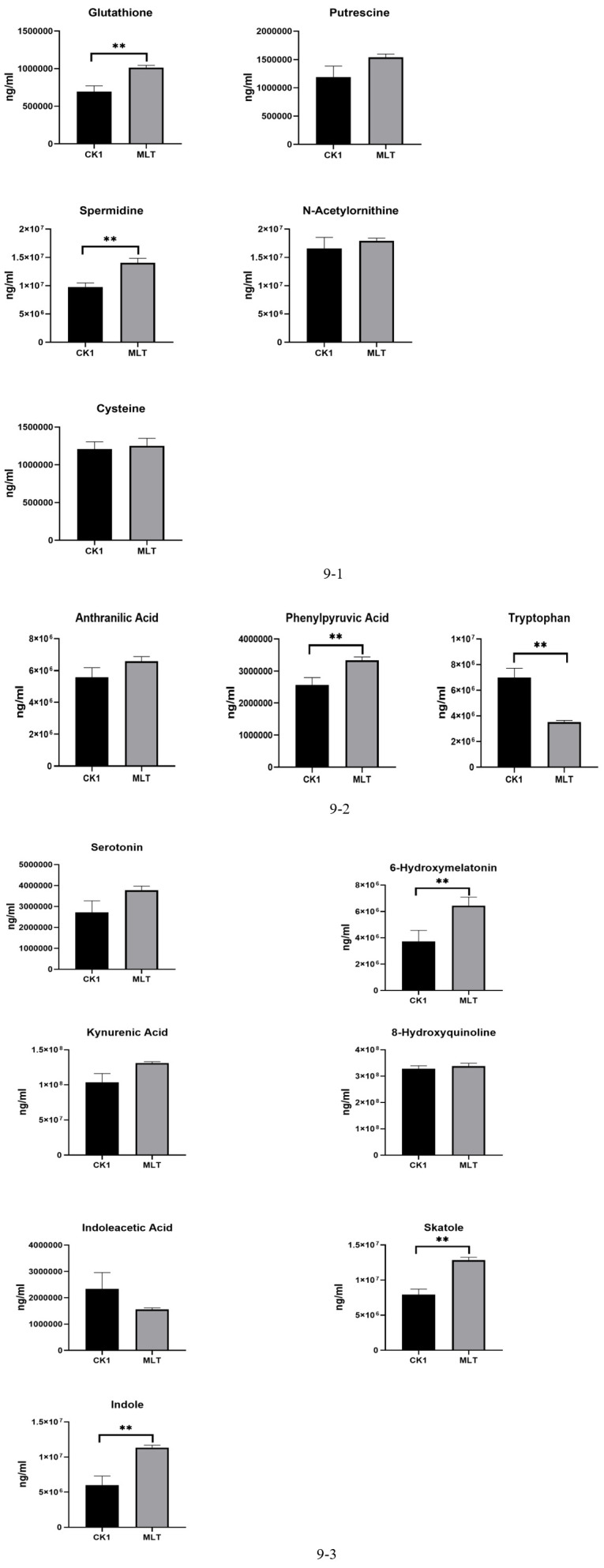
(1) Metabolites in the glutathione pathway. * means significant correlation. (2) Metabolites in the phenylalanine, tyrosine, and tryptophan biosynthesis pathway. * means significant correlation. (3) Metabolites in the tryptophan metabolic pathway. * means significant correlation.

In the glutathione metabolic pathway (Fig. 9-1) of glutathione, putrescine, spermidine, N-acetylornithine, and cysteine, the contents of glutathione and spermidine increased significantly (*P* < 0.05). In the biosynthesis pathway of phenylalanine, tyrosine, and Try (Fig. 9-2), the contents of metabolites of 6-aminobenzoic acid, phenylpyruvic acid, and Try were detected, and the content of phenylpyruvic acid was significantly increased (*P* < 0.05). In the Try metabolic pathway (Fig. 9-3), the metabolites serotonin, kynurenine, quinolinic acid, indoleacetic acid, indole-3-methyl, indole, and 6-hydroxymelatonin were detected, and the contents of indole, indole-3-methyl, and 6-hydroxymelatonin metabolites were increased significantly (*P* < 0.05).

#### Correlation of microbiota, metabolites, and enzymes

**Fig 10:**
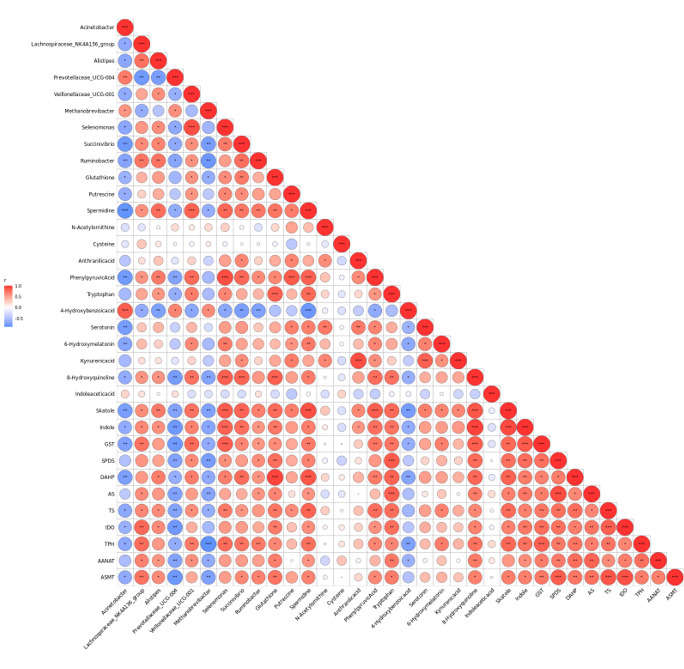
Correlation analysis of microorganisms, metabolites, and enzymes.

The rank correlations of Spearman (Fig. 10) among the different rumen microbiota, rumen metabolites, and enzymes revealed that *Lachnospiraceae_NK4A136_group* was related to GST, IDO, TPH, and ASMT. *Prevotellaceae_UCG-004* had a significant negative correlation with metabolites such as phenylpyruvate, quinolinic acid, indole-3 methyl, indole, GST, SPDS, AS, TS, IDO, AANAT, and ASMT (*P* < 0.01). *Veillonellaceae_UCG-001* was positively correlated with spermidine, phenylpyruvate, quinolinic acid, indole-3-methyl, GST, and TPH (*P* < 0.01). *Methanobrevibacter* was negatively correlated with indole-3-methyl, quinolinic acid, SPDS, AS, TPH, and ASMT (*P* < 0.01). *Selenomonas* had a significant positive correlation between six metabolites and GST, TS, and TPH (*P* < 0.01). There was a significant positive correlation between *Succinivibrio* and six metabolites and DAHP and TPH (*P* < 0.01).

## DISCUSSION

### Effects of MLT treatment on fermentation parameters and VFA content *in vitro*

MLT is a rhythmic hormone but is easily degraded. Therefore, MLT concentration in the CK0 group was measured as a reference for the MLT group. NH_3_-N, pH, MCP, and VFA in the rumen of ruminants reflect rumen environmental conditions, nitrogen supply and utilization, microbial population, NH_3_-N uptake and utilization capacity, and microbial digestion and metabolism (20, 21). Rumen environment indicators (NH_3_-N, pH, MCP, VFA, etc.) are important factors for evaluating both rumen and ruminant health. The results showed that although the MLT concentration in the CK0 group decreased after 24 h of fermentation in the rumen juice, the MLT degradation rates in the CK1 and MLT groups were significantly higher than those in the CK0 group (*P <* 0.05), which may be due to the use of MLT by some microorganisms. In addition, there were no significant differences in pH, NH_3_-N, and MCP between the MLT group and the CK1 group in this study, but the NH_3_-N concentration decreased and the MCP increased. The reason may be that MLT reduces the decomposition of nitrogen-containing substances by rumen microorganisms, resulting in a decrease in the concentration of NH_3_-N. Subsequently, the microorganisms use the undecomposed nitrogen-containing substances of rumen microorganisms to synthesize MCP. VFA is the main energy supplier in the synthesis process. VFA in the rumen is mainly produced by microbial fermentation of carbohydrates in the diet, which can provide energy up to 70%–80% (22). The results showed that the addition of MLT increased the concentrations of propionic acid, isobutyric acid, butyric acid, valeric acid, and TVFA in the fermentation broth, and the acetic acid to propionic acid ratio was <2.5. The results also indicated that the addition of MLT changed the rumen fermentation type, made the rumen fermentation develop to propionic acid type, provided more energy for the body, promoted microbial fermentation, and provided more energy for the MCP synthesis process (23). For ruminants, nutrients in the diet were degraded by microorganisms in the rumen, producing a large amount of hydrogen, which can be used to synthesize energy substances such as methane, propionic acid, and ATP. This may explain why the concentration of NH_3_-N decreased while the concentration of VFA increased.

### Effects of MLT treatment on the structure of rumen microbial flora in *in vitro* fermentation

The rumen of ruminants is home to a wide variety of microorganisms. The microbial flora structure and relative abundance were in a dynamic equilibrium state, which had a positive impact on maintaining the stability of the rumen environment, digestion, nutrient absorption, and metabolism (24). The relationship between intestinal microbial population changes and MLT had gradually become a research hotspot. By comparing colitis mice with MLT-treated colitis mice, Zhu et al. (25) found that the structure of intestinal flora was changed, in which *Firmicutes* increased and *Proteobacteria* decreased. In this study, it was found that after adding MLT, the dominant bacteria in the experimental group and the CK1 group were *Bacteroidetes*, *Firmicutes,* and *Proteobacteria*. This result was consistent with the results of a previous study (26). The genome of *Bacteroidetes* had a large number of carbohydrate-active enzyme genes, which can use a variety of dietary soluble polysaccharides. The metabolites of *Bacteroides* are mainly acetate and propionate (27). *Firmicutes* were rich in genes related to biosynthesis and membrane transport, and their metabolite was mainly butyrate (28). Most *Proteobacteria* were not pathogenic, with the exception of a few bacteria, such as *Escherichia coli* and *Shigella. Proteobacteria* can encode a large number of protein and carbohydrate metabolic genes and play an important role in maintaining the anaerobic environment of the intestine (29). VFA was mainly affected by anaerobic microorganisms such as *Lachnospiraceae*, *Ruminobacter,* and *Bacteroides* in the intestine, which played an active role in antioxidant, anti-inflammatory, anti-tumor activities, regulating gene expression and intestinal flora balance and improving intestinal functions (30, 31). A study had found that VFA can be used as a signal molecule to inhibit intracellular histone deacetylase by activating extracellular G-protein-coupled receptors and may help to reduce intestinal inflammation (32). In this study, we found that the addition of MLT significantly increased the relative abundance of VFA-producing bacteria such as *Ruminobacter*, *Akkermansia,* and *Lachnospiraceae_NK4A136_group*, which played a positive role in inhibiting inflammation and improving intestinal health. Therefore, in this study, the increase in propionic acid, isobutyric acid, butyric acid, and TVFA may be related to the increase in these microorganisms.

At the genus level, the genera of the dominant bacteria in the MLT group and the CK1 group were *Acinetobacter. Acinetobacter* belonged to γ Moraceae of the *Proteus* class, which is widely distributed in nature (33). Most of them were opportunistic pathogens, which posed a serious threat to human and animal health and also to the environment (34). *Acinetobacter* can be transmitted through air circulation and animal movement, resulting in cross-transfer between the animal body, farm fecal pollution, and environmental equipment (35, 36). The results of our study showed that the experimental animals may have been infected with *Acinetobacter* but did not exhibit obvious signs and symptoms. This may be related to the infection time and the stress resistance of Hu sheep, but it needs to be further verified and studied. The content of *Acinetobacter* in the rumen fluid of the experimental group decreased significantly, indicating that MLT had a pronounced inhibitory effect on *Acinetobacter*. The reasons for the reduction of *Acinetobacter* in fermentation broth may be as follows. (i) MLT may work via a mechanism to directly inhibit *Acinetobacter.* (ii) The significant increase in beneficial antibacterial *Ruminobacter*, *Lachnospiraceae_NK4A136_group,* and *Akkermansia* may inhibit the relative abundance of *Acinetobacter*, in which *Lachnospiraceae* had an antagonistic effect on pathogens (37). In addition, we speculate that MLT may be more effective in the inhibition of *Acinetobacter* in organisms because MLT can improve the body’s antioxidant capacity and increase the body’s immune regulation. However, how MLT inhibits *Acinetobacter* and its specific regulatory mechanism need to be further studied.

### Effects of MLT treatment on rumen microbial metabolites in *in vitro* fermentation

In this study, the addition of MLT increased the relative contents of some rumen microbial metabolites, such as skatole, glutathione (GSH), spermidine, indole, phenylpyruvic acid, and prostaglandin, but the contents of 4-hydroxybenzoic acid and alanine were relatively reduced.

GSH was a bioactive peptide that is widely present in cells. In the bacterial domain, GSH is mainly found in Gram-negative and some Gram-positive bacteria, including *Cyanobacteria* and *Proteobacteria* (38). GSH had a variety of important physiological functions in organisms, especially in maintaining a suitable redox environment. GSH can prevent the oxidation of proteins by forming disulfide bonds, and it can also act as a substrate of glutathione peroxidase (GPx) to inhibit lipid peroxidation, thereby playing an antioxidant role (39). In cells, GSH can be used as a source of amino acids (40, 41). MLT plays a direct role in inhibiting the activation of the NF-κB signaling pathway and contributes to the reduction of inflammation (42). In addition, MLT is an essential regulator of GSH (43). It can reduce GSH depletion by regulating c-glutamylcysteine synthetase and GSH reductase (44, 45). In this study, the addition of MLT increased the expression of c-glutamylcysteine synthase and GSH reductase, thus increasing the content of GSH.

Spermidine is a polyamine compound existing in all eukaryotes (46), which played an important role in maintaining DNA stability and regulating cell growth and proliferation, differentiation, apoptosis, tissue regeneration, and antioxidation and anti-inflammatory processes (47). A study had found that spermidine can pass through the NF-κB signaling pathway, reducing the expression levels of pro-inflammatory factors such as IL-6 and TNF-α in BV2 microglia (48). A variety of microbial components, including lipopolysaccharide (LPS), can participate in the regulation of the immune system by MLT through the NF-κB signaling pathway (49, 50). Based on the above studies and our results, it can also be speculated that microorganisms, MLT, and spermidine may work through the NF-κB signaling pathway to reduce the expression of inflammatory factors. At the same time, MLT had a certain regulatory relationship with microorganisms. Our study found that MLT can increase the contents of spermidine. These may indicate that MLT, microorganisms, and spermidine had a certain regulatory relationship, but the specific mechanism needs to be further explored. Spermidine in organisms is an important intracellular cationic compound. It can interact with negatively charged macromolecules (such as nucleic acids, proteins, and phospholipids) (51, 52) and participate in a variety of cellular functions, such as virulence (53), acid resistance (54), biofilm formation (55), and free radical scavenging (56). Previous studies had found that exogenous spermidine enhanced the sensitivity of β-lactam in *Acinetobacter* (57). Exogenous polyamines can reduce GSH levels in *Acinetobacter*, which may be linked to spermidine-mediated β-lactam sensitivity (57). This also explained why spermidine and glutathione were significantly related to *Acinetobacter* in the current study. At the same time, pathway analysis showed that MLT metabolism might be regulated by rumen microbes through the GSH metabolism pathway. Whether MLT has the potential to inhibit the growth of *Acinetobacter* through the GSH metabolic pathway needs further investigation.

Indole is produced by tryptophanase catalyzing L-Try with pyridoxal 5′-phosphate as a coenzyme, along with pyruvate and ammonia (58). Indole can decrease the expression of two key proteins (p60, IκBα) of NF-κB, which potentially promote the immunomodulatory effects of indole (59). Indole can also upregulate the gene expression of Ptgs2 in the liver, potentially promoting the production of prostaglandin E2, which has immunomodulatory properties (59). These studies are consistent with the changing trend of indole and prostaglandin contents in this study. Trp was the precursor of MLT. It, along with its metabolites, is closely related to MLT anabolism. Trp, endogenous Trp metabolites (tyrosine, serotonin, and MLT), and Try products of bacterial metabolism (indole, indole acid, and tryptamine) had far-reaching effects on intestinal microbial composition and microbial metabolism (11). Trp is decarboxylated to tryptamine under the combined action of *Clostridium* fusiform and *Ruminococcus*. Indole acetic acid can be decarboxylated into skatole by *Lactobacillus*, *Clostridium,* and *Bacteroides* in the gut (1).

Trp mainly had two endogenous metabolic pathways in the intestine: kynurenine pathway (KP) and serotonin pathway. Among them, 95% of Trp is degraded to kynurenine, xanthine, nicotinic acid, 4-hydroxybenzoic acid, and so on through the KP pathway, while 1%–2% of Trp is degraded to serotonin and MLT through the serotonin pathway, which also involved TPH (60). A study has found that bacterial Trp metabolites can affect intestinal barrier integrity and immune cell metabolites through the aromatic hydrocarbon receptor (AHR) (61). Microorganisms can directly affect Trp metabolism and the level of its metabolites, which can target AHR and regulate immune function (62). AHR is a cytoplasmic ligand-activated transcription factor that mediates the metabolism of xenobiotics and is a key regulator of immunity and inflammation (63). The ligands of AHR include xanthine acid, indole, and skatole. Increased AHR ligands contribute to intestinal immunity (64). AHR had been found to activate lymphocyte 3 (ILC3) to produce IL-22, which modulates antimicrobial peptide release and maintains immune response and microbial balance by modulating microbial composition (65).

**Fig 11:**
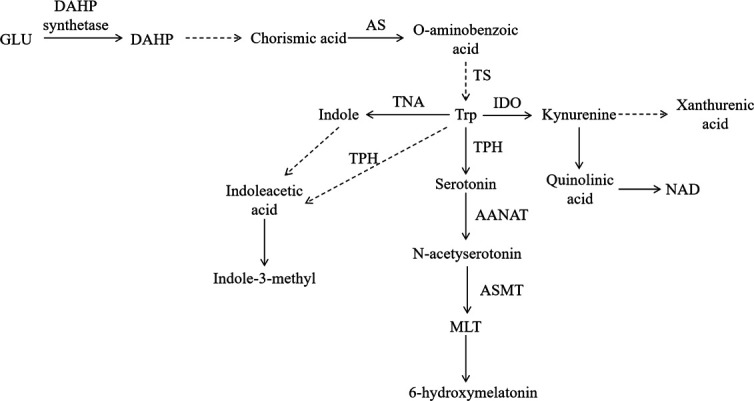
Pathway of MLT promoting tryptophan production and metabolism of rumen microorganisms. The solid line represents a one-step biochemical reaction; the dotted line represents an indirect reaction.

By Kyoto Encyclopedia of Genes and Genomes (KEGG) enrichment of differential metabolites, we found that metabolites were mainly enriched in the GSH metabolic pathway and two pathways of phenylalanine, tyrosine, and Try biosynthesis. Among them, by classifying the pathways of differential metabolites, it was found that it is also related to Trp metabolism. In our study, the significantly different metabolites 4-hydroxybenzoic acid, TPH, indole, and xanthine were related metabolites of the Trp metabolic pathway. Therefore, it can be speculated that MLT may regulate intestinal microorganisms through the Trp metabolic pathway (Fig. 11). Meanwhile, the functional analysis of pathways also supported this analytical view. The enrichment analysis of significantly different metabolites had a significant impact on the biosynthesis of phenylalanine, tyrosine, and Try in the main signaling pathway. Therefore, we speculated that microorganisms may synthesize phenylalanine, tyrosine, and Try after the addition of MLT. After the synthesis of Trp, Trp metabolism continued to generate relevant metabolites. Among them, skatole had a significant positive correlation with *Selenomonas* and *Succinivibrio*, while skatole could only be produced by microorganisms. Therefore, we further speculated that MLT may mediate the “Trp biosynthesis and metabolic pathway” through *Selenomonas* and *Succinivibrio*, but it needs further study.

### Analysis of the mechanism and regulatory pathway of MLT acting on rumen microbial receptors

**Fig 12:**
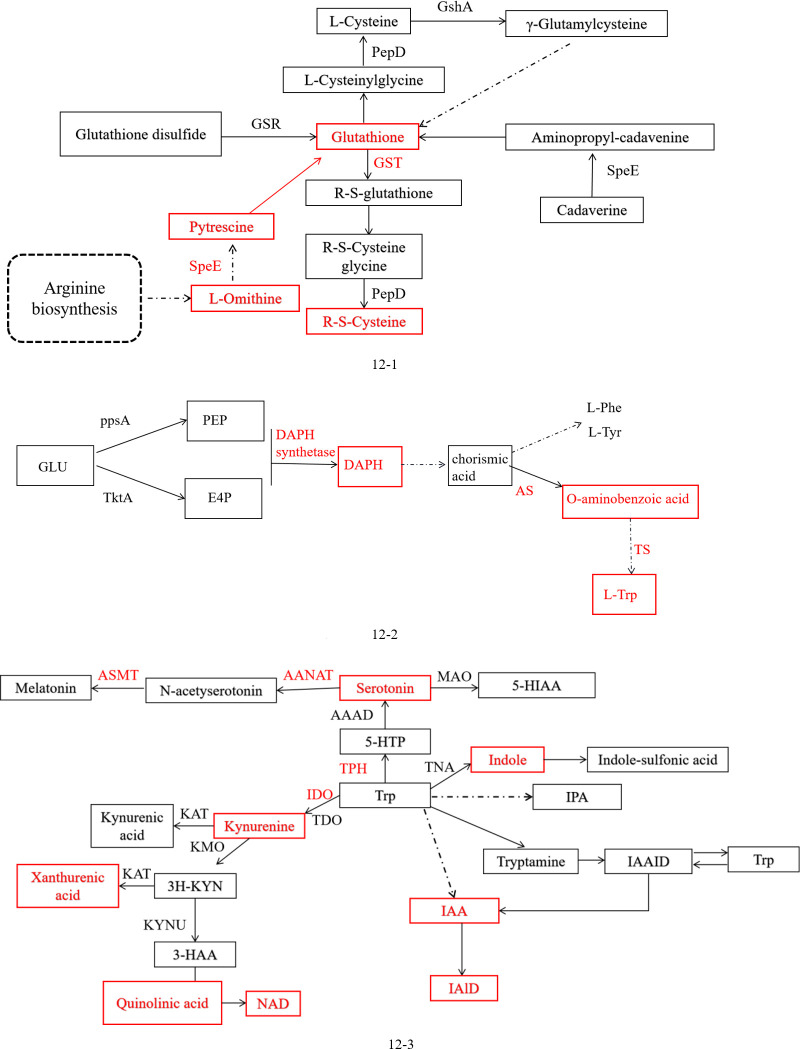
(1) Glutathione metabolism. (2) Phenylalanine, tyrosine, and tryptophan biosynthesis pathway. (3) Tryptophan metabolic pathway.

In the results, we found that the addition of MLT significantly increases the activities of the enzymes GST and SPDS in the glutathione pathway and the contents of glutathione and spermidine metabolites (*P* < 0.05). Combined with the results of the apparent reduction in harmful bacteria, it can be speculated that MLT may depend on the regulation of the glutathione metabolic pathway to alter the flora structure. In the results of our study, from the arginine-glutathione pathway, it is known that L-ornithine synthesizes putrescine (spermidine) via spermidine synthase, followed by the production of GSH. Then, glutathione generated R-S-cysteine under the action of glutathione-S-transferase, as shown in red color in Fig. 12-1. The results of this study are similar to a previous report (66). Recent studies have found that the addition of MLT can enhance the cardioprotective effects of mice exposed to carbon tetrachloride and change the activity of glutathione metabolic (glutathione peroxidase, S-transferase, and reductase) enzymes (67). Liu et al. (68) showed that the addition of MLT can alleviate the oxidative stress of IMD-treated cucumber and increase the activity of glutathione-S-transferase by regulating the ascorbic acid glutathione cycle.

The biosynthesis of aromatic amino acids usually existed in microorganisms and plants. The synthetic pathways are divided into the central metabolic pathway, common pathway, and branch pathway. It used glucose (Glu) as raw material and condenses to form 3-deoxyribonucleic acid under the joint action of erythritose (E4P) and phosphoenolpyruvate-α-arabinoheptanoic acid-7-phosphate (DAHP), producing phenylalanine, tyrosine, and Try, respectively. In the pathway, DAHP was the first-rate limiting enzyme in the central metabolic pathway, and AS and TS were the key enzymes of Try synthesis in the branched amino acid pathway and were regulated by Try feedback inhibition (69). Our results showed that the activities of DAHP, AS, and TS increased significantly in the experimental group (*P* < 0.05). In addition, the metabolites DAHP, o-aminobenzoic acid, phenylpyruvate, and Try were detected, and phenylpyruvate and Try were significantly different (*P* < 0.05). Therefore, it was speculated that MLT may regulate rumen microorganisms through aromatic amino acid biosynthesis (the red in Fig. 12-2).

In the Try metabolic pathway (Fig. 12-3), IDO participates in the metabolism of the kynurenine pathway and plays an important role in immune tolerance. IDO is expressed in most cells, but the expression level is low. However, the expression of IDO will increase when the body is inflamed or infected. IDO can also reduce the Try concentration of T cells and play a negative regulatory role in immune regulation (70). In this study, it was found that the dominant bacteria of rumen microorganisms in experimental animals were harmful *Acinetobacter*, indicating that the body may have been infected with *Acinetobacter*. The body was infected, which induced the enhancement of IDO activity and increased the degradation of Try. In Try metabolism, kynurenine metabolites such as kynurenine, xanthine, quinolinic acid, and NAD were detected, suggesting that the addition of MLT may regulate microorganisms through the Try-kynurenine metabolic pathway. TPH is a key enzyme in the “Trp-5-HTP-serotonin” pathway. A previous study has found that microorganisms can synthesize serotonin through the “Trp-tryptamine-serotonin” pathway (71). In our study, we also found that in this pathway, the activities of the key enzymes TPH, AANAT, and ASMT were significantly enhanced, and the metabolites Try, MLT, serotonin, and 6-hydroxymelatonin were also detected (*P <* 0.05) (Fig. 12-3). Indole can protect bacteria from antibiotics and balance the inflammation of the mammalian intestine with other Trp derivatives by regulating flagella synthesis, virulence factor expression, or intestinal bacteria (72, 73). This seems to explain the significant decrease in the relative abundance of *Acinetobacter* after the addition of MLT. Therefore, we speculated that the increase in MLT may regulate the metabolism of Trp, significantly increase the contents of indole and indole-3-methyl (skatole) in the “Trp-indole” metabolic pathway, and then reduce the relative abundance of *Acinetobacter*.

Microbiota can directly or indirectly modulate the host endogenous metabolism of Trp, and its changes may have significant effects on microbial proliferation and microbiota diversity. The levels of Trp and 5-hydroxytryptamine (5-HT) were significantly increased in germ-free mice lacking gut microbiota (74, 75). This may be attributed to gut microbial metabolites, such as short-chain fatty acids or Trp-derived indole metabolites, which promote 5-HT production in the gut to regulate the level of Trp (76, 77). The gut microbiota of humans and mice promoted the expression of TPH and production of 5-HT in the colon through stimulation of entero chromaffin cells (ECs) by short-chain fatty acids (78). In our study, propionic acid, isobutyric acid, butyric acid, valeric acid, and TVFA concentrations were significantly increased. Therefore, the significant expression of TPH may be due to the short-chain fatty acids produced by gut microbes to promote the expression of TPH. It can be inferred that MLT may first regulate gut microbes, leading to changes in microecological structure and microbial fermentation products, and then regulate metabolic pathways. However, the exact regulatory mechanism needs to be determined by more experiments.

### Conclusion

This study revealed the regulation of MLT on rumen microbial fermentation parameters, microbial community structure, and rumen fermentation metabolites *in vitro*. First, our study found an important point: MLT can inhibit the abundance of *Acinetobacter*, which may be due to the increase in beneficial bacteria and the anti-inflammatory and immune effects of MLT. Second, MLT treatment changed the structure of rumen flora, in which anaerobic microorganisms such as *Lachnospiraceae*, *Ruminococcus,* and *Bacteroidetes* accelerated the utilization of carbohydrates in the fermented diet, further increased the content of metabolite VFA, and changed the rumen fermentation state, thereby promoting the generation of metabolites such as TPH and 5-HT and regulating metabolic pathways. Specifically, *Lachnospiraceae_NK4A136_group*, *Prevotellaceae_UCG-004*, *Veillonellaceae_UCG-001*, *Methanobrevibacter,* and *Selenomonas* regulated the “arginine-glutathione pathway” and increased the contents of glutathione and spermidine metabolites under the action of GST and SPDS enzymes. The pathway of the “aromatic amino acid biosynthesis-Try generation branch” was regulated by *Prevotellaceae_UCG-004*, *Methanobrevibacter*, *Succinivibrio,* and *Selenomonas* under the action of DAHP, AS, and TS enzymes, which significantly increased the content of phenylpyruvate and Try, and then Try was metabolized. Under the action of TPH and IDO enzymes, *Lachnospiraceae_NK4A136_group*, *Prevotellaceae_UCG-004*, *Veillonellaceae_UCG-001*, *Methanobrevibacter*, *Selenomonas*, *Succinivibrio,* and *Ruminobacter* regulated the “Try-kynurenine metabolism” pathway and significantly increased the contents of xanthine acid and quinolinic acid metabolites. Under the action of AANAT and ASMT enzymes, *Lachnospiraceae_NK4A136_group*, *Prevotellaceae_UCG-004*, and *Methanobrevibacter* can increase the content of 6-hydroxymelatonin by regulating the “Try-tryptamine-serotonin” pathway. MLT may regulate Try production and metabolic pathways through rumen microorganisms, which in turn affect the metabolism of MLT.

## Data Availability

Metagenomics data of sequencing are available in the Genome Sequence Archive in the BIG Data Center (https://bigd.big.ac.cn/), and the accession number is CRA011077.
